# 

**Published:** 1840-01

**Authors:** 


### CONTENTS OF NO. I.

ORIGINAL CCOMMUNICATIONS.

The Mortality of Infants, by H. MILLER, M.D. - - - - 3
A Case of Fatal Abscess of the Kidney, by L. POWELL, M.D. 25
On Secale Cornutum, &c., by Dr. HIGGASON. - - - 29

REVIEWS AND BIBLIOGRAPHICAL NOTICES.

Lectures on the Theory and Practice of Physic, by the late David Hosack, M.D. &c. &c. - - - - - - - 33
Ingalls on Scarlatina, - - - - - - - - - 55
Pennock and Moore on the Action of the Heart, - - - 56

SELECTIONS FROM AMERICAN AND FOREIGN JOURNALS.

On the treatment of Lateral Curvatures of the Spine, by JULES GUERIN, M.D. - - - - - - - - - 59
Case of a woman delivered of five children, - - - - 60
Of Malformation, - - - - - - - - - ib.
Galvanic Obstetric Forceps, - - - - - - - 61
On Hydrated Peroxyde of Iron as an Antidote to Arsenic, - ib.
Urinary diseases, - - - - - - - - 64

Case of Intus-susceptio, &c. - - - - - - - 67
Bleeding in Affections of the Brain, - - - - - 68
History of the last Illness of M. Broussais, - - - ib.
Origin of Medical Journals, - - - - - - 71
On the Vesicating properties of the Weevil, - - - 72
Dysentery, - - - - - - - - - - 74
Extirpation of a part of a Rib for Neuralgia, - - - 77

ORIGINAL INTELLIGENCE.

Our Enterprize, - - - - - - - - - 79
Yellow Fever in the South West, - - - - , 80
Climate of Tropical Florida, - - - - - - 82
Death from Mercurial Fumigation, - - - - - 84
Milk Sickness, - - - - - - - - ib.
Translation of Trosseau and Bellog, on Laryngitis, - - 85
Gross' Pathological Anatomy, - - - - - - ib.
Scarlatina Simplex, - - - - - - - - ib.
Louisville Medical Institute, - - - - - - 86
To Correspondents, - - - - - - - - ib.

